# Using a discrete choice experiment to estimate individual preferences to medicate cancer-related symptoms with cannabis

**DOI:** 10.1186/s42238-026-00392-1

**Published:** 2026-01-26

**Authors:** Helen McTaggart-Cowan, Adam J.N. Raymakers, Sara Izadi-Najafabadi, Colene Bentley

**Affiliations:** 1 Population Health Sciences, BC Cancer Research Institute, Vancouver, Canada; 2 Faculty of Health Sciences, Simon Fraser University, Burnaby, Canada

**Keywords:** Canada, Cancer, Cannabis, Discrete choice experiment, Preferences, Cancer survivorship

## Abstract

**Background:**

In October 2018, Canada legalized non-medical cannabis, increasing accessibility nationwide. Although a separate medical cannabis framework exists, many individuals with cancer may now obtain cannabis through non-medical channels rather than through the medical system. This shift raises questions about how legalization has influenced patients’ attitudes and preferences regarding the use of cannabis to manage cancer-related symptoms and treatment side effects. We aimed to determine individuals’ preferences for using cannabis as a complementary therapy in cancer survivorship.

**Methods:**

Members of a Canadian research panel completed a discrete choice experiment (DCE) to assess preferences for medicinal cannabis. Respondents completed 12 choice sets, each consisting of two health states described by seven attributes identified from previous qualitative work, as well as an opt-out option. The seven attributes were cannabis effectiveness, ability to perform everyday activities, chance of unwanted side effects, opinions of family and friends about cannabis, doctor’s opinion of cannabis, access to cannabis, and out-of-pocket cost. Each attribute had three or four levels. DCE responses were analyzed using an error-component mixed logit regression model to estimate the relative attribute importance and willingness-to-pay.

**Results:**

The dataset included 1,089 respondents who completed at least one choice set. Of these, 61.5% (*n* = 670) reported no experience with cancer, and 35.5% (*n* = 387) had some experience with cannabis. Analysis of the DCE responses demonstrated that respondents preferred the effective management of cancer symptoms and the ability to perform everyday activities. Respondents expressed disutility for unwanted side effects resulting from cannabis use.

**Conclusion:**

To our knowledge, this is the first study to elicit general population preferences for medicinal cannabis in the context of cancer survivorship. Insights from these preferences may inform policy and priority-setting to support equitable access to medical cannabis for symptom management improved quality of life among individuals with cancer.

**Supplementary Information:**

The online version contains supplementary material available at 10.1186/s42238-026-00392-1.

## Introduction

People living with cancer often experience a wide range of symptoms and treatment-related side effects that persist well beyond the completion of active treatment [1, 2]. These include both physical and psychological challenges such as chemotherapy-induced nausea and vomiting, pain, fatigue, anorexia, insomnia, anxiety, and depression [3, 4]. When inadequately managed, these symptoms can substantially impair quality of life, daily functioning, and overall recovery.

Cannabis or cannabinoid-based products – hereafter, referred to as medicinal cannabis – have attracted growing interest as complementary therapies for managing cancer-related side effects [5–8]. Cannabinoids, such as Δ9-tetrahydrocannabinol (THC) and cannabidiol (CBD) exert their effects through the endocannabinoid system, primarily via CB1 and CB2 receptors involved in modulating pain, inflammation, appetite, mood, and emesis [6, 9]. Evidence suggests that THC can reduce chemotherapy-induced nausea and vomiting and stimulate appetite, while both THC and CBD may alleviate pain, anxiety, and sleep disturbances [10, 11]. However, effectiveness varies across individuals, and cannabis is typically considered in relation to a broader spectrum of available symptom-management options, including opioids, antiemetics, anxiolytics, antidepressants, and non-pharmacologic interventions [12].

Although medical cannabis has been legally available in Canada since 2001, access has historically occurred through federally authorized mail-order programs or unlicensed medical dispensaries [13]. Despite more than two decades of medical use, there remain no standardized clinical guidelines regarding dosing, product selection, or therapeutic use in oncology. The legalization of non-medical (recreational) cannabis legalization in 2018 further expanded access but did not resolve these clinical gaps [12] and retail store staff are prohibited from offering health-related advice. This has left many individuals to make decisions with limited medical guidance [14]. This evolving landscape has blurred distinctions between medical and non-medical use, and may impact how people with and without cancer experience perceive and access cannabis for symptom management.

Preferences reflect the value individuals place on various aspects of health and healthcare [15]. From a health economics perspective, they can be understood through the concept of utility, which is a measure of satisfaction or benefit a person derives from a certain set of healthcare options and their associated trade-offs [16]. Discrete choice experiments (DCEs) are a well-established method for quantifying such preferences and allow researchers to estimate how individuals value particular treatment attributes and the trade-offs they are willing to make [17].

In Canada’s publicly funded healthcare system, understanding public preferences helps ensure that healthcare decisions reflect societal values and promote equitable access. DCEs also enable decision-making under a ”veil of ignorance”, in which individuals make choices without knowing their future health status, an approach that supports fair and efficient allocation of healthcare resources [18]. Therefore, the aim of the study was to elicit general population preferences for medicinal cannabis in the context of cancer survivorship following the legalization of non-medical cannabis in Canada [19]. These insights will inform healthcare decisions, clinical practice, and policy development to better reflect the diverse values and needs of the Canadian population.

## Methods

We used a DCE to elicit individual preferences for medicinal cannabis use in the context of cancer survivorship. DCEs are widely applied to explore patient, healthcare provider, and policy-maker preferences for different characteristics of health-related products, services, and health interventions [20–23]. A DCE was chosen over other preference elicitation methods because it allows respondents to evaluate hypothetical scenarios and make explicit trade-offs between characteristics, thereby enabling direct measurement of utility.

DCEs assume goods and services can be described by characteristics (called attributes), each defined by a set of levels that represent realistic variations. Attributes should be relevant, understandable, and capable of influencing choice. Levels should be realistic and distinct without being dominant, ensuring that respondents would face meaningful trade-off across attributes [16]. This design enables estimation of preference-based utility values from observed choices. Using experimental design techniques, combinations of attribute levels form scenarios were used to construct a series of choice tasks from combinations of the attribute levels using the package “dcreate” in Stata Version 16.1 (StataCorp; College Station, USA)[24]. A D-optimal approach was used to maximize the statistical efficiency of the model [25].

Each choice task consists of two or more scenarios, and respondents are asked to choose the scenario they most prefer [24]. Respondents were asked to imagine themselves as cancer survivors deciding whether to use cannabis to help manage cancer-related symptoms. Information of all attributes were provided before the DCE to ensure understanding. An introductory preamble and a short animated explainer video described the task [26]; the video script was also available as text for accessibility (Appendix A). Respondents were informed that they will be comparing features of the hypothetical scenarios to select the option that they prefer the most.

The development of the DCE in this analysis was done in four stages: identification of attributes and levels; experimental design; data collection; and data analysis and interpretation. The qualitative work used to develop the attributes and levels for the DCE has been published elsewhere [26]. The remaining stages are the focus of this study.

### Identification of attributes and levels

Attribute and level development was informed by previous qualitative research [26] and guided conceptually by the Theory of Planned Behaviour (TPB) framework [27]. The TPB posits that behavioural intentions are shaped by behavioural beliefs (advantages and disadvantages of an action), normative beliefs (perceived social influences), and control beliefs (perceived facilitators and barriers).

We identified seven attributes associated with the use of medicinal cannabis to inform the DCE design based on our previous qualitative work [26] and guided by research team discussion to ensure that they were sensitive to the Canadian context, modifiable to produce levels and trade-offs, and amenable to policy intervention. Three attributes reflected behavioural beliefs: effectiveness, ability to perform everyday activities, and chance of unwanted side effects. Two attributes reflected normative beliefs: support from family and friends and support from physicians. Two attributes reflected control beliefs: access to cannabis and out-of-pocket cost. Each attribute included multiple levels (Table 1). Guided by TPB but refined using patient language from previous qualitative work [14, 26], attributes and levels were reviewed and pre-tested to ensure that no single level was unambiguously preferable, thereby minimizing dominance and encouraging realistic trade-offs.

The access to cannabis attribute described different ways individuals might obtain medicinal cannabis. Levels were defined as prescription obtained from my doctor to represent current authorization process; prescription from a cannabis store to present access facilitated through a specialized clinic license to provide medical cannabis guidance; obtained from a neighbourhood dispensary to represent retail access through a licensed recreational store; and obtained from another source to represent any other means of accessing cannabis outside of medical authorization or license retail settings. The cost levels reflected plausible out-of-pocket costs associated with cannabis use in Canada. We reviewed published and grey literature on medical and recreational cannabis pricing; both medical and recreational markets were considered when determining a realistic range [28–30]. The chance of experiencing unwanted side effects attribute referred to the likelihood of side effects commonly reported with cannabis use. During the preamble, side effects were described as including fatigue, dizziness, dry mouth, impaired concentration, increased heart rate, and the sensation of being “high”.

**Table 1:** List of attributes and levels

Theory of Planned Behaviour Construct	Attributes	Levels
Behavioural beliefs	Effectiveness of cannabis	• Cancer symptoms are managed some of the time • Cancer symptoms are managed most of the time • Cancer symptoms are managed all the time
	Everyday activities	• Performed some of the time • Performed most of the time • Performed all the time
	Chance of getting unwanted side effects	• Low • Moderate • High
Normative beliefs	Opinions of my family and friends about cannabis	• Does not support my cannabis medication • Somewhat supports my cannabis medication • Supports my cannabis medication
	My doctor’s opinions about cannabis	• Does not support my cannabis medication • Somewhat supports my cannabis medication • Supports my cannabis medication
Control beliefs	Cannabis access	• Prescription obtained from my doctor • Prescription obtained from a cannabis clinic • Obtained from a neighbourhood dispensary • Obtained from another source
	My monthly out-of-pocket cost	• $50 • $100 • $250 • $750

### Experimental design

**Fig. 1:**
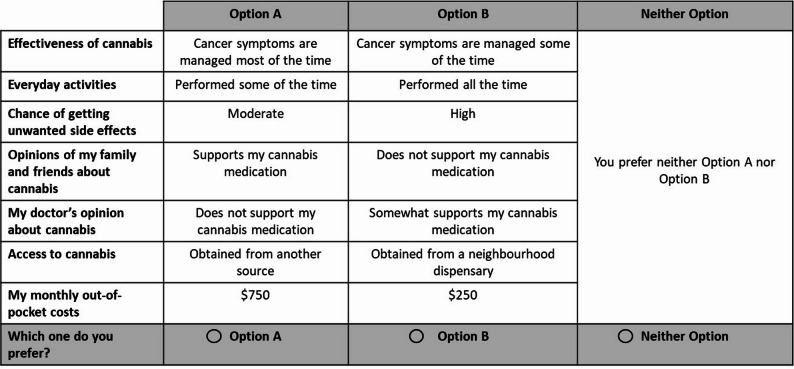
Example of choice task

A scenario consisted of one level from each attribute. The full factorial design (i.3., 3^5^ × 4^2^ = 3,888 possible scenarios) was reduced using a fractional factorial experimental design to ensure efficiency and respondent feasibility. The resulting 48 scenarios were split into two blocks of 24 scenarios (12 choice tasks per respondent). Each choice task included two cannabis therapy scenarios (Option A and Option B) and an opt-out alternative, allowing respondents to indicate a preference for neither option. An example of a choice task is presented in Fig. 1.

### Data collection

Data were collected by Mustel Research [31] from an online Canadian panel of adults aged 18 years and older. Quota sampling ensured alignment with national census distributions for age, sex, and province/territory of residence [32]. Respondents were recruited using regional quotas (Atlantic, Central, Prairies, West, and North) to align with the Canadian population distribution. Although analyses are presented by provinces and territories to enhance interpretability, recruitment was based on these broader regions. Respondents could donate a small honorarium to a charity of choice. There is no consensus on the determination of appropriate sample sizes in DCEs [33] but the final sample exceeded the suggested 20 responses per choice set to estimate reliable models [34]. The panel provider applied standard data quality controls before releasing the final dataset.

Respondents completed the following survey components: (i) basic screening demographics for quota sampling; (ii) DCE valuation task; (iii) attitudinal questions about cannabis use (these results are not provided here); and (iv) sociodemographic characteristics. Ethics approval was obtained from the Research Ethics Board at BC Cancer (H19-01489).

### Data analysis

DCE responses were analyzed using an error-component mixed logit regression model [35]. Analyses included all respondents who completed at least one choice task [36]. Respondents who completed at least one choice task were retained because each choice provides independent information to the mixed logit model, and excluding partial completers may reduce statistical efficiency and introduce selection bias [22, 34, 37]. All categorical attributes were effects-coded; cost was treated as a continuous variable. An alternative specific constant (ASC) represented a preference for either cannabis option versus the opt-out. Cost was fixed to facilitate estimation.

Coefficient magnitudes reflected the relative importance of attribute-level utilities. Marginal rates of substitution between attribute levels and cost yielded willingness-to-pay (WTP) estimates. WTP provides information not only about the valuation of the bundle of characteristics we are trying to evaluate but also about the trade-offs between attributes that may not be traditionally measured in monetary terms [25]. The differences in the WTP estimates may be dependent on individuals’ cannabis experience (i.e., cannabis experience vs. no cannabis experience) and cancer experience (i.e., cancer experience vs. no cancer experience). We used the delta method to generate 95% confidence intervals (CIs) for WTP estimates [16]. A negative WTP estimate indicates how much an individual is willing to pay to avoid something not preferred. All analyses were conducted using Stata version 13.1 [24].

## Results

### Study population

Respondents from the Canadian general population consented to participate in the study (*n* = 1,992). Of these, 689 were removed because they did not complete any screening questions (*n* = 31), were under age (*n* = 17), the demographic quota was fulfilled (*n* = 641), or they quit before reaching the actual DCE choice tasks (*n* = 214). The final analysis included *n* = 1,089: 51 completed at least one choice set and 1,038 completed all choice sets. Table 2 presents the characteristics of the study population. *Post ho*c analysis showed that there were statistically significant differences between the respondents in our dataset and the population in terms of age groups and province/territory of residence. The majority of the study sample self-reported to be white (68.4%), reported having received a university education (53.4%) and described their health as very good or excellent (55.7%). Many respondents reported experience with cancer, with 61.5% having cared for someone undergoing or who had undergone cancer treatment, and 8.4% having received cancer treatment themselves, either currently or in the past. Approximately one third of respondents reported having experience with cannabis.

**Table 2:** Study population characteristics

**Characteristics**	**Count (%)**	**Population value (%)**	**Χ**^2^ statistic	*P* value
Sex assigned at birth				
Female	543 (49.9%)	51.5	1.17	0.28
Self-identified gender				
Male gender	969 (49.4%)			
Female gender	973 (49.6%)			
Gender diverse	7 (0.4%)			
Prefer not to answer	12 (0.6%)			
Age (years)			33.1	<0.001
18-29	195 (9.8%)	12.9		
30-39	383 (19.2%)	13.1		
40-49	273 (13.7%)	13.1		
50-59	362 (18.2%)	15.1		
60-69	391 (19.6%)	12.1		
70-79	310 (15.6%)	6.9		
>80	78 (3.9%)	4.3		
Province/territory of residence			61.9	<0.001
BC	251 (12.8%)	13.6		
AB	232 (11.8%)	11.2		
SK	50 (2.6%)	3.0		
MB	67 (3.4%)	3.5		
ON	755 (38.5%)	38.2		
QC	387 (19.7%)	23.4		
NB	36 (1.8%)	2.2		
NS	64 (3.3%)	2.7		
PEI	12 (0.6%)	0.4		
NL	96 (4.9%)	1.5		
YT	2 (0.1%)	0.1		
NWT	1 (0.05%)	0.1		
NU	8 (0.4%)	0.1		
Education				
No certificate, diploma or degree	12 (1.2%)			
High school	109 (10.5%)			
Apprenticeship or trades	43 (4.2%)			
College	222 (21.5%)			
University degree less than bachelor’s degree	87 (8.4%)			
University degree bachelor’s degree or greater	552 (53.4%)			
Prefer not to answer	9 (0.9%)			
Marital status				
Never legally married	248 (24.0%)			
Legally married	501 (48.5%)			
In common-law relationship	119 (11.5%)			
Separated but still legally married	28 (2.7%)			
Divorced	80 (7.7%)			
Widowed	41 (4.0%)			
Prefer not to answer	17 (1.6%)			
Employment				
Self employed	86 (8.3%)			
Paid employment	575 (55.6%)			
Full-time student or at school	27 (2.6%)			
Retired	266 (25.7%)			
Unemployed	29 (2.8%)			
Looking after family or home	17 (1.6%)			
Disability	17 (1.6%)			
Other	6 (0.6%)			
Prefer not to answer	11 (1.1%)			
Cultural group				
Indigenous	12 (1.2%)			
White	713 (68.4%)			
Black	25 (2.4%)			
Latin	15 (1.4%)			
South Asian	49 (4.7%)			
East and Southeast Asian	154 (14.8%)			
Middle and West Asian	24 (2.3%)			
Other	21 (2.0%)			
Prefer not to answer	40 (3.8%)			
General Health Question				
Excellent	159 (15.4%)			
Very good	416 (40.3%)			
Good	362 (35.0%)			
Fair	83 (8.0%)			
Poor	13 (1.3%)			
Cancer experience				
Currently undergoing cancer treatment	16 (1.5%)			
Have completed cancer treatment	75 (6.9%)			
Cared for someone who is undergoing or has undergone cancer treatment	671 (61.5%)			
No direct cancer experience	327 (30.1%)			
Use of cannabis for medical or non-medical reasons				
Yes	394 (36.2%)			
No	665 (61.1%)			
Unsure	12 (1.1%)			
Prefer not to answer	18 (1.7%)			
Cannabis frequency				
Did not use cannabis	194 (48.1%)			
Once during 4 weeks	40 (9.9%)			
Twice during 4 weeks	30 (7.4%)			
Once a week	26 (6.5%)			
2-3 times a week	46 (11.4%)			
Everyday	31 (7.7%)			
Multiple times a day	25 (6.2%)			
Prefer not to answer	11 (2.7%)			

### Preference estimates

Respondents demonstrated positive utility for the management of cancer symptoms and for the ability to perform everyday activities, although not all attribute levels were monotonic or statistically significant (Table 3). Respondents also preferred support from family members and friends when indicating their medicinal cannabis preference. Disutility was expressed for the chance of unwanted side effects, indicating that respondents strongly preferred to avoid them. Participants also expressed disutility regarding the source of cannabis when compared to receiving a doctor’s prescription.

**Table 3:** Results of error-component mixed logit regression

Attribute and level	Part-worth utility, mean^Ɨ^	Part-worth utility, SD^ǂ^
Effectiveness of cannabis		
Cancer symptoms managed some of the time	Ref	
Cancer symptoms managed most of the time	−1.611	1.888*
Cancer symptoms managed all of the time	4.187*	1.849*
**Ability to perform everyday activities**		
Everyday activities performed some of the time	Ref	
Everyday activities performed most of the time	3.329*	6.495*
Everyday activities performed all of the time	1.801	0.585
**Chance of unwanted side effects**		
Low	Ref	
Moderate	−2.991*	6.134*
High	−12.522*	1.524
**Opinions from family and friends about cannabis**		
No support	Ref	
Somewhat supportive	4.443*	2.646*
Supportive	4.465*	3.536*
**My doctor’s opinions about cannabis**		
No support	Ref	
Somewhat supportive	0.107	2.117*
Supportive	0.903	0.179
**Cannabis access**		
Doctor’s prescription	Ref	
Cannabis clinic	−4.021*	1.417*
Neighbourhood dispensary	−1.954	2.406
Other sources	1.073	3.498*
**Cost**	−0.0029	

* *p* < 0.05; R^2^ = 0.287

^Ɨ^The mean part-worth utility value indicates the utility associated with each attribute level. Part-worth utility values can be summed to indicate the overall utility of a product

^ǂ^The SD is an estimated model parameter (with its own standard error indicating statistical significance)

Table 4 presents the WTP estimates stratified by respondents’ experience with cannabis and by their experience with cancer. When comparing the WTP estimates by cannabis experience, respondents without cannabis experience were willing to pay more for medicinal cannabis to better manage cancer-related symptoms and improve their ability to perform everyday activities compared to those with cannabis experience. They were also willing to pay more to have greater support from their doctor regarding cannabis use. Respondents with cancer experience had higher and statistically significant WTP estimates for the attribute levels compared to respondents without cancer experience. All respondents reported that they were willing to pay to avoid a high chance of unwanted side effects compared to a low chance. Respondents without cancer experience reported the highest WTP to avoid the highest chance of unwanted side effects at $838/month.

**Table 4:** Willingness-to-pay estimates stratified by individuals’ cannabis and cancer status

Attribute	Willingness-to-pay estimate ($/month) (mean, 95% CI)			
	Individuals with cannabis experience (*n* = 387)	Individuals without cannabis experience (*n* = 702)	Individuals with cancer experience (*n* = 409)	Individuals without cancer experience (*n* = 680)
Effectiveness of cannabis				
Cancer symptoms managed some of the time	Ref	Ref	Ref	Ref
Cancer symptoms managed most of the time	179 (−230 to 588)	235 (120 to 350)*	184 (137 to 230)*	419 (−2126 to 1288)
Cancer symptoms managed all of the time	61 (−317 to 439)	275 (165 to 386)*	243 (197 to 288)*	175 (−997 to 1348)
**Ability to perform everyday activities**				
Everyday activities performed some of the time	Ref	Ref	Ref	Ref
Everyday activities performed most of the time	381 (−109 to 870)	228 (106 to 350)*	174 (124 to 224)*	420 (−27 to 867)
Everyday activities performed all of the time	287 (−145 to 719)	319 (199 to 439)*	304 (254 to 355)*	193 (−206 to 592)
**Chance of unwanted side effects**				
Low	Ref	Ref	Ref	Ref
Moderate	−109 (−474 to 257)	−9 (−114 to 96)	−127 (−171 to −82)*	−75 (−417 to 267)
High	−789 (−1385 to −193)*	−457 (−587 to −327)*	−551 (−608 to −495)*	−838 (−1413 to −265)*
**Opinions from family and friends about cannabis**				
No support	Ref	Ref	Ref	Ref
Somewhat supportive	−133 (−527 to 262)	89 (−12 to 191)	121 (78 to 163)*	3 (−357 to 351)
Supportive	213 (−173 to 599)	26 (−81 to 133)	98 (53 to 142)*	276 (−111 to 662)
**My doctor’s opinions about cannabis**				
No support	Ref	Ref	Ref	Ref
Somewhat supportive	171 (−224 to 565)	184 (79 to 289)*	175 (131 to 218)*	520 (78 to 962)*
Supportive	231 (−190 to 652)	260 (146 to 374)*	215 (169 to 261)*	616 (90 to 1143)*
**Cannabis access**				
Doctor’s prescription	Ref	Ref	Ref	Ref
Cannabis clinic	449 (−115 to 1013)	235 (95 to 375)*	150 (91 to 208)*	524 (−27 to 1075)
Neighbourhood dispensary	300 (−242 to 842)	222 (75 to 369)*	183 (121 to 245)*	317 (−208 to 841)
Other sources	434 (−94 to 962)*	442 (299 to 584)*	273 (217 to 328)*	715 (134 to 1297)*

* *p* < 0.05

## Discussion

This analysis reports on the elicitation of individual preferences for medical cannabis in the context of cancer-related symptom management. Overall, respondents expressed preferences for the different attribute levels presented in the DCE. The magnitude of these preferences differed according to respondents’ experiences with cannabis and cancer.

Consistent with the theoretical framework of the TPB, the attributes reflecting behavioural beliefs had a significant impact on respondents’ preferences. Respondents expressed the greatest disutility for unwanted side effects. This finding aligns with our earlier qualitative work [14], in which participants raised concerns about side effects, particularly unknown interactions between cancer therapies and medicinal cannabis. In this study, the WTP estimates should be interpreted with caution, as they are specific to the decision-making context. Not all attribute levels followed a monotonic gradient. For example, some respondents valued “symptom management most of the time” more than “all of the time”. Such non-monotonicity may indicate response heuristics, limited differentiation between similar high levels, or cognitive burden when evaluating complex trade-offs. Future qualitative work could explore how respondents interpret subtle level differences. Further, the levels assigned to the cost attribute can influence the WTP estimates for other attributes in a DCE [38]. In our study, the presented WTP estimates need to be interpreted with care as there is great variance in the data, as noted by the large SDs, with the only attributes describing effectiveness of cannabis and doctor’s opinion were statistically significant. Additional work may be warranted to see if respondents were applying heuristics when making their decisions.

The DCE results also revealed that support from family and friends were more influential than support from a doctor. This finding is partially consistent with our earlier qualitative study, where participants described normative beliefs regarding the need for approval from their medical team and their social network when deciding whether to manage their cancer-related symptoms with cannabis [14]. Interestingly, the strength of preference for doctor support was weaker than for family and friend support. Moreover, there appeared to be a mismatch between respondents’ stated preference for doctor’s support and their preference for accessing cannabis through a cannabis clinic, neighbourhood dispensary or another source rather than through a doctor’s prescription. This discrepancy warrants further exploration. Including both social support attributes in the DCE reflects guidance emphasizing that DCE instruments should capture preferences beyond immediate health outcomes [39]. However, the process of evaluating all medicinal cannabis attributes simultaneously may have encouraged respondents to make trade-offs that are distinct from the way individuals would articulate beliefs according to TPB constructs in qualitative interviews. This finding may also reflect the shifting normative attitudes following cannabis legalization, with increasing reported comfort in certain social settings [40].

Previous work exploring preferences for medicinal cannabis has highlighted the distinction between intrinsic or extrinsic product attributes [41]. This study primarily focused on extrinsic attributes, as they were identified through qualitative work [14, 26]. Intrinsic product attributes (e.g., cannabinoid ratio, cannabis species or strain, and product quality) were not identified as salient in our earlier work [14, 26]. Earlier studies suggested that individuals may have preferences regarding the route of cannabis administration [41], although these studies were not cancer specific. In the current study, the chance of unwanted side effects was the only intrinsic attribute included.

This study had several limitation. First, respondents were recruited from an online research panel. Although quota sampling was used to approximate representativeness by age, sex, and region of residence, the final sample differed from the Canadian population in terms of age and region. Respondents were also primarily white and were highly educated, and only a small proportion reported experience with cannabis or cancer, only a small number were currently undergoing or had completed cancer treatment. The extent to which these factors may have influenced preference estimates is unknown. Future work should examine preference heterogeneity across diverse groups, including individuals actively receiving cancer treatment and cancer survivors.

Second, the online survey format introduces potential non-coverage bias, as the decision to participate in the study is at the discretion of the individuals and we do not have means to knowing about those who do not participate [42]. Despite data quality checks by the panel provider, undetected low-quality or automated (“bot”) responses cannot be fully excluded. The English-only survey may also have excluded individuals with limited English proficiency. Further, a small number of participants completed only one choice task. Although each completed task contributes independent preference information and was therefore retained in the analysis, partial completion may introduce additional measurement error at the individual level.

Third, although we implemented an explainer video [26] and provided definitions for the attributes, there remains a possibility of misinterpretation. This is particularly relevant for the “chance of unwanted side effects” attribute. The DCE described the likelihood of experiencing unwanted side effects but did not specify which side effects were being considered. Individuals may have interpreted this differently. For example, weighing a mild euphoric “high” against more distressing physiological effects such as increased heart rate or dizziness. Such variation in interpretation may have influenced the trade-offs respondents were willing to make relative to cancer-related symptoms. Future research should explore whether specifying the type or severity of side effects improves clarity without increasing respondent burden. Similarly, terms such as “prescription from a cannabis clinic” or “neighbourhood dispensary” could have been interpreted differently by some respondents. Although the attributes and levels were derived from qualitative work and followed established guidelines for DCE content validity, the potential for heterogeneity in interpretation remains.

Finally, while the attributes and levels were derived from established theory and qualitative work, attributes important to some individuals may still have been omitted. As legislation and models of cannabis access continue to evolve in Canada, periodic reassessment of relevant attributes will be important to ensure that DCE instruments remain contextually accurate.

The findings from this study contribute to the growing literature on preferences for medical cannabis, particularly in the context of cancer-related symptom management [41]. Preference elicitation provides one important form of evidence, alongside clinical benefit (including effectiveness and safety), cost-effectiveness, and feasibility of adoption that can inform effective policy and priority-setting decisions regarding medicinal cannabis for people with cancer for the management of cancer-related symptoms in Canada.

## Supplementary Information

Supplementary Material 1

## Data Availability

The datasets used and/or analyzed during the current study are available from the corresponding author on reasonable request.

## References

[1] Shapiro CL. Cancer survivorship. N Engl J Med. 2018;379(25):2438–50. 30575480 10.1056/NEJMra1712502

[2] Link C, DeIure A, Watson L. Understanding the post-treatment concerns of cancer survivors with five common cancers: exploring the Alberta results from the pan-Canadian transitions study. Curr Oncol. 2022;29(4):2662–80. 35448192 10.3390/curroncol29040218 PMC9026535

[3] Schmidt ME, Goldschmidt S, Hermann S, Steindorf K. Late effects, long-term problems and unmet needs of cancer survivors. Int J Cancer. 2022;151(8):1280–90. 35657637 10.1002/ijc.34152

[4] Faithfull S, Greenfield D. Cancer survivor late-effects, chronic health problems after cancer treatment: what’s the evidence from population and registry data and where are the gaps? Curr Opin Support Palliat Care. 2024;18(1):55. 38170192 10.1097/SPC.0000000000000692

[5] Steele G, Arneson T, Zylla D. A comprehensive review of cannabis in patients with cancer: availability in the USA, general Efficacy, and safety. Curr Oncol Rep. 2019;21(1):10. 30707319 10.1007/s11912-019-0757-7

[6] National Academies of Sciences, Engineering, and, Medicine H, Division M, Board on Population Health and Public Health Practice, Committee on the Health Effects of Marijuana: An Evidence Review and Research Agenda. The Health Effects of Cannabis and Cannabinoids: The Current State of Evidence and Recommendations for Research [Internet]. Washington (DC): National Academies Press (US); 2017 [cited 2023 July 10]. (The National Academies Collection: Reports funded by National Institutes of Health). Available from: http://www.ncbi.nlm.nih.gov/books/NBK423845/

[7] Blake A, Wan BA, Malek L, DeAngelis C, Diaz P, Lao N, et al. A selective review of medical cannabis in cancer pain management. Ann Palliat Med. 2017;6(Suppl 2):S215–22. 28866904 10.21037/apm.2017.08.05

[8] Victorson D, McMahon M, Horowitz B, Glickson S, Parker B, Mendoza-Temple L. Exploring cancer survivors’ attitudes, perceptions, and concerns about using medical cannabis for symptom and side effect management: a qualitative focus group study. Complement Ther Med. 2019;47:102204. 31779995 10.1016/j.ctim.2019.102204

[9] Leinen ZJ, Mohan R, Premadasa LS, Acharya A, Mohan M, Byrareddy SN. Therapeutic potential of cannabis: A comprehensive review of current and future applications. Biomedicines. 2023;11(10):2630. 37893004 10.3390/biomedicines11102630 PMC10604755

[10] Cannabis and Cannabinoids in Adults With Cancer. ASCO Guideline | Journal of Clinical Oncology [Internet]. [cited 2025 Oct 24]. Available from: 10.1200/JCO.23.02596 10.1200/JCO.23.02596 PMC11730458 38478773

[11] Bathula PP, Maciver MB. Cannabinoids in treating Chemotherapy-Induced nausea and Vomiting, Cancer-Associated Pain, and tumor growth. Int J Mol Sci. 2023;25(1):74. 38203245 10.3390/ijms25010074 PMC10779013

[12] National Academies of Sciences E, Division H, Practice M, on PH B, Agenda PH. C on the HE of MAER and R. Therapeutic Effects of Cannabis and Cannabinoids. In: The Health Effects of Cannabis and Cannabinoids: The Current State of Evidence and Recommendations for Research [Internet]. National Academies Press (US); 2017 [cited 2025 Oct 24]. Available from: https://www.ncbi.nlm.nih.gov/books/NBK425767/28182367

[13] Shim M, Nguyen H, Grootendorst P. Lessons from 20 years of medical cannabis use in Canada. PLoS One. 2023;18(3):e0271079. 36952564 10.1371/journal.pone.0271079 PMC10035846

[14] McTaggart-Cowan H, Bentley C, Raymakers A, Metcalfe R, Hawley P, Peacock S. Understanding cancer survivors’ reasons to medicate with cannabis: a qualitative study based on the theory of planned behavior. Cancer Med. 2021;10(1):396–404. 33068314 10.1002/cam4.3536 PMC7826491

[15] Bridges JFP. Stated preference methods in health care evaluation: an emerging methodological paradigm in health economics. Appl Health Econ Health Policy. 2003;2(4):213–24. 15119540

[16] Regier DA, Peacock SJ, Pataky R, van der Hoek K, Jarvik GP, Hoch J, et al. Societal preferences for the return of incidental findings from clinical genomic sequencing: a discrete-choice experiment. CMAJ. 2015;187(6):E190–7. 25754703 10.1503/cmaj.140697 PMC4387060

[17] Hauber AB, González JM, Groothuis-Oudshoorn CGM, Prior T, Marshall DA, Cunningham C, et al. Statistical methods for the analysis of discrete choice experiments: a report of the ISPOR conjoint analysis good research practices task force. Value Health. 2016;19(4):300–15. 27325321 10.1016/j.jval.2016.04.004

[18] Neumann PJ, Russell LB, Siegel JE, Prosser LA, Krahn M, Mandelblatt JS et al. Using Cost-Effectiveness Analysis in Health and Medicine: Experiences since the Original Panel. In: Neumann PJ, Ganiats TG, Russell LB, Sanders GD, Siegel JE, editors. Cost-Effectiveness in Health and Medicine [Internet]. Oxford University Press; 2016 [cited 2023 July 10]. p. 0. Available from: 10.1093/acprof:oso/9780190492939.003.0001

[19] Canada H. Understanding the Access to Cannabis for Medical Purposes Regulations [Internet]. 2016 [cited 2024 May 27]. Available from: https://www.canada.ca/en/health-canada/services/publications/drugs-health-products/understanding-new-access-to-cannabis-for-medical-purposes-regulations.html

[20] Ryan M, Gerard K, Amaya-Amaya M,Using Discrete Choice Experiments to Value Health and Health Care [Internet]. Dordrecht: Springer Netherlands;, Bateman IJ, editors. The Economics of Non-Market Goods and Resources; vol. 11). Available from: http://link.springer.com/10.1007/978-1-4020-5753-3

[21] Clark MD, Determann D, Petrou S, Moro D, de Bekker-Grob EW. Discrete choice experiments in health economics: a review of the literature. Pharmacoeconomics. 2014;32(9):883–902. 25005924 10.1007/s40273-014-0170-x

[22] de Bekker-Grob EW, Ryan M, Gerard K. Discrete choice experiments in health economics: a review of the literature. Health Econ. 2012;21(2):145–72. 22223558 10.1002/hec.1697

[23] Soekhai V, de Bekker-Grob EW, Ellis AR, Vass CM. Discrete choice experiments in health economics: Past, present and future. PharmacoEconomics. 2019;37(2):201–26. 30392040 10.1007/s40273-018-0734-2 PMC6386055

[24] StataCorp. Stata statistical software: release 16. College station. TX: StataCorp LLC; 2019.

[25] Kuhfeld WF, Tobias RD, Garratt M. Efficient experimental design with marketing-research applications. J Mark Res. 1994;31:243–64.

[26] Bentley C, Izadi-Najafabadi S, Raymakers A, McTaggart-Cowan H. Qualitative research informing a preference study on selecting cannabis for cancer survivor symptom management: design of a discrete choice experiment. Patient. 2022;15(4):497–507. 35132605 10.1007/s40271-021-00567-3 PMC9197893

[27] Ajzen I. The theory of planned behavior. Organ Behav Hum Decis Process. 1991;50(2):179–211.

[28] Government of Canada SC. Cannabis consumer prices [Internet]. 2018 [cited 2025 Oct 28]. Available from: https://www150.statcan.gc.ca/t1/tbl1/en/tv.action?pid=1810021101

[29] Mahamad S, Hammond D. Retail price and availability of illicit cannabis in Canada. Addict Behav. 2019;90:402–8. 30530299 10.1016/j.addbeh.2018.12.001

[30] Mahamad S, Wadsworth E, Rynard V, Goodman S, Hammond D. Availability, retail price and potency of legal and illegal cannabis in Canada after recreational cannabis legalisation. Drug Alcohol Rev. 2020;39(4):337–46. 32291811 10.1111/dar.13069

[31] Mustel Group | Mustel Group [Internet]. [cited 2023 July 10]. Available from: https://mustelgroup.com/

[32] Canada S. 2016 Census of Population [Internet]. 2021 [cited 2023 July 10]. Available from: https://www12.statcan.gc.ca/census-recensement/2016/index-eng.cfm

[33] Norman R, Viney R, Brazier J, Burgess L, Cronin P, King M, et al. Valuing SF-6D health States using a discrete choice experiment. Med Decis Mak. 2014;34(6):773–86. 10.1177/0272989X13503499 24025661

[34] Lancsar E, Louviere J. Conducting discrete choice experiments to inform healthcare decision making: a user’s guide. Pharmacoeconomics. 2008;26(8):661–77. 18620460 10.2165/00019053-200826080-00004

[35] Train KE. Discrete Choice Methods with Simulation [Internet]. 2nd ed. Cambridge: Cambridge University Press; 2009 [cited 2023 July 10]. Available from: https://www.cambridge.org/core/books/discrete-choice-methods-with-simulation/49CABD00F3DDDA088A8FBFAAAD7E9546

[36] Janssen EM, Hauber AB, Bridges JFP. Conducting a discrete-choice experiment study following recommendations for good research practices: an application for eliciting patient preferences for diabetes treatments. Value Health. 2018;21(1):59–68. 29304942 10.1016/j.jval.2017.07.001

[37] Hole AR. A discrete choice model with endogenous attribute attendance. Econ Lett. 2011;110(3):203–5.

[38] Drummond MF, Sculpher M, Claxton K, Stoddart GL, Torrance GW. Methods for the economic evaluation of health care programmes. Fourth edition. Oxford New York: Oxford University Press; 2015. 445 p.

[39] Ryan M. Using conjoint analysis to take account of patient preferences and go beyond health outcomes: an application to in vitro fertilisation. Soc Sci Med. 1999;48(4):535–46. 10075178 10.1016/s0277-9536(98)00374-8

[40] Winfield-Ward L, Hammond D. Social norms for cannabis use after nonmedical legalization in Canada. Am J Prev Med. 2024;66(5):809–18. 38128676 10.1016/j.amepre.2023.12.013

[41] Gething K, Erku D, Scuffham P. Stakeholders’ decisions and preferences for the provision and use of medicinal cannabis: a scoping review. Cannabis Cannabinoid Res. 2023;8(6):986–98. 10.1089/can.2022.0115 36888538

[42] Fricker RD, London England EC1Y 1SP United Kingdom. Sampling Methods for Web and E-mail Surveys. In: The SAGE Handbook of Online Research Methods [Internet]. 1 Oliver’s Yard, 55 City Road, : SAGE Publications, Ltd; 2008 [cited 2023 July 11]. pp. 195–216. Available from: https://methods.sagepub.com/book/the-sage-handbook-of-online-research-methods/n11.xml

