# Supplementary material for: Using a discrete choice experiment to estimate individual preferences to medicate cancer-related symptoms with cannabis
Source: J Cannabis Res. 2026 Jan 26;8:30. doi: 10.1186/s42238-026-00392-1 (PMC12918061; doi:10.1186/s42238-026-00392-1)
Supplement: Supplementary file 1 — Supplementary Material 1 [file 42238_2026_392_MOESM1_ESM.docx]

**Appendix A: Transcript of explainer video**

In Canada, nearly 1 in 2 people will develop cancer in their lifetime, and about 1 in 4 will die of cancer. The good news is that due to advances in science, 63% of patients will survive cancer for 5 years or longer.

Many cancer survivors experience long-term effects of cancer, like nerve pain, insomnia, fatigue, and anxiety about the cancer returning. These symptoms can last for many years, and have a negative impact on survivors’ quality of life.

Cancer survivors manage their cancer symptoms in different ways, like:

- Taking prescription medicines
- Exercising, meditating, and counselling
- And taking cannabis, or marijuana, which is available with or without a prescription

Cannabis is derived from the cannabis plant. It can be used for medical and recreational purposes.

- Cannabis comes in different forms: it can be smoked, eaten, or taken as an extract (like an oil).
- Some cancer survivors take cannabis to help manage the long-term effects of cancer.
- Consuming cannabis can produce other mental and physical effects, including:
  - Relaxation
  - Euphoria
  - Increased appetite
  - And difficulty concentrating

There are many things to consider when deciding to manage cancer symptoms with cannabis. They include:

- How well does cannabis work for my cancer symptoms?
- Will taking cannabis enable me to perform my usual day-to-day activities, or will I experience unwanted side effects?
- Is it important that others support my decision to manage my cancer symptoms with cannabis?
- Where can I buy cannabis, and how much would it cost?
- In the survey you are about to complete, you will be asked to imagine that you are a cancer survivor faced with the decision of choosing cannabis to help treat your cancer-related symptoms.
- The survey will show you hypothetical “decision scenarios”.
- These are used to understand what features of cannabis medication are important to you. Your job is to compare the features and then select the option you prefer the most.


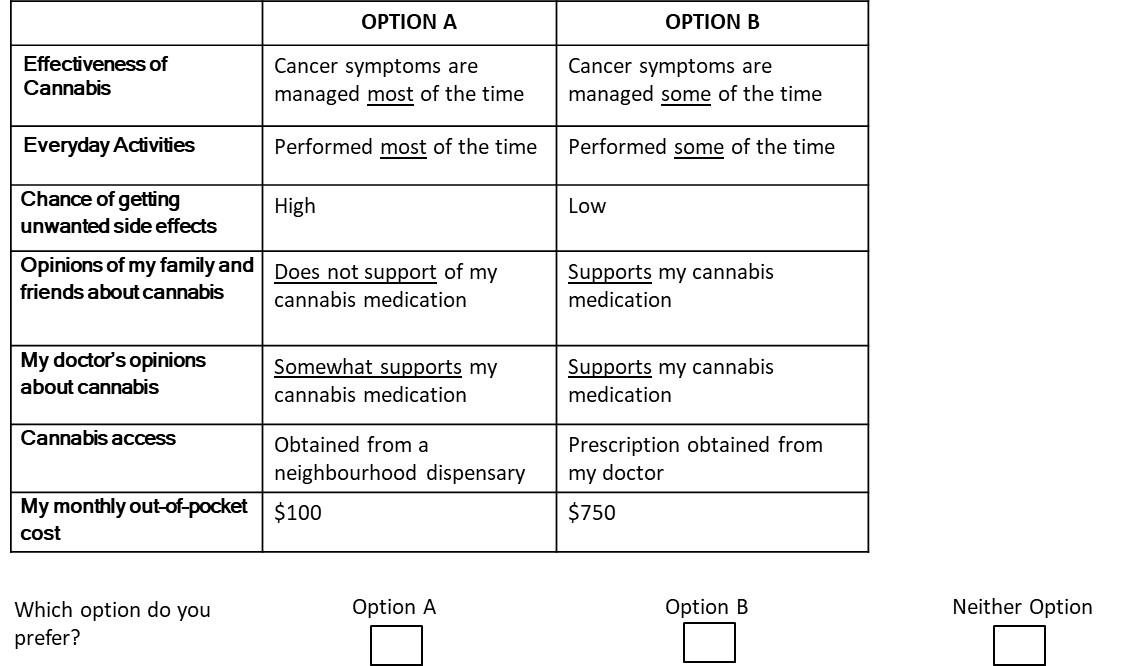


The different features of cannabis are listed on the left-hand column. The cannabis features apply to both Option A, and Option B.

You will notice there are slight differences in the features between Option A and Option B. For example, the effectiveness of cannabis for Option A states “your cancer symptoms are managed most of the time”; for Option B, it states “your cancer symptoms are managed some of the time”. Each feature will have slight differences between Option A and Option B.

At the end of the scenario, you will be asked to select which option you prefer.

Now you are ready to take the survey. The survey will show you similar decision scenarios and ask you to select which option you prefer. There are no right or wrong answers. Make your choices based on what you would like, not what you think other people would like.

Your job is to: (i) pay close attention to the different combinations of cannabis features; (ii) consider all the features presented; (iii) make trade-offs between them; and (iv) select the set of features you think is best for you.

You will be asked to repeat this decision process 12 times. While the questions may seem repetitive, the values within the features will keep changing, so it is important you read the specific features of each treatment carefully.
